# microRNA Bioinformatics

**DOI:** 10.3390/cells11223677

**Published:** 2022-11-18

**Authors:** Y-h. Taguchi

**Affiliations:** Department of Physics, Chuo University, Tokyo 112-8551, Japan; tag@granular.com

Firstly, I apologize for the delayed publication of this Special Issue in the form of a book title. The newest submission was made more over two years ago (24 November 2020). Thus, the content of this book is somewhat outdated.

Since the identification of microRNA (miRNA) as one of the functional non-coding RNAs, several studies have been conducted. Despite the appearance of more functional families of non-coding RNAs, miRNAs still remain key players in a wide range of research issues.

Reflecting the versatile functions of miRNAs, this volume covers a wide range of topics. Mohammadi-Dehcheshmeh et al. [1] attempted to identify miRNAs that can target SARS-CoV-2 transcripts and found that a rare human miRNA, hsa-MIR-5004-3p, can target these transcripts. Unfortunately, this finding has not been experimentally verified.

Reflecting the increased interest in the interaction between SARS-CoV-2 and human miRNAs, as mentioned previously, Fernandes et al. [2] investigated the general interactions between MicroRNAs and Mammarenaviruses. In contrast to the above findings [1], which indicates the possibility of human miRNAs targeting SARS-CoV-2, conversely, there is a possibility that viruses make use of miRNAs to progress their infection to the hosts. It is indicated that viruses might control various signaling pathways by controlling miRNA expression.

miRNAs are important not only in mamarean, but also in other vertebrates. Bovolenta et al. [3] established that the miRNA database is specific to one non-popular species: Nile Tilapia. Although Nile Tilapia cannot be regarded as a typical research target of molecular biology, such as human, mouse, yeast, fly and C. elegance, more than two thousand papers addressing Nile Tilapia are listed in PubMed, one of the major biological paper databases. Although it has not yet been cited by any papers, this kind of study is nonetheless valuable.

Other than relatively new topics about miRNAs, studies on more traditional topics are also included in this volume. Martinez-Gutierrez et al. [4] attempted to identify critical miRNAs in breast cancer. The importance of miRNAs in cancers is well known. This study found that many miRNAs might play a critical role in breast cancer in a group-oriented manner. Other than cancers, miRNAs are also known to be critical in many diseases. Hromadnikova et al. [5] found that the expression of several altered miRNAs is critical in childish gestational diabetes mellitus. In addition to studies that focus on individual diseases, the study of general methods that can identify the relation between diseases and miRNAs is also essential. Ha at al [6] established an improved method that makes use of network analyses. Interactions between diseases and miRNAs, as well as miRNAs and host genes, are important. Zeidler et al. [7] investigated the interaction between host genes and intergenic miRNAs. The integration between disease association and host-gene miRNAs is often valuable to research. Osone and Yoshida [8] reasoned the interaction between miRNAs and diseases using hydrogen bonding sites in RNA–RNA interactions. Sometimes, integrated analyses that make use of networks can benefit the study of specific diseases. Uddin et al. [9] employed this approach to investigate colon cancer. Zhang et al. [10] used deep learning to tackle a traditional problem: the association of miRNAs and disease. Biomarkers using miRNAs are important and promising research topics. Khavari and Cairns [11] reviewed recent efforts in this direction, specifically targeting schizophrenia. Finally, this volume includes one special article that targets data issues [12]. Xia et al. developed a database that stores gene expression, including miRNAs.

In conclusion, this volume successfully collates a wide range of topics about miRNAs, ranging from the application of new machine learning to both traditional and novel topics, such as SARS-CoV-2.
